# Estimating valence from the sound of a word: Computational, experimental, and cross-linguistic evidence

**DOI:** 10.3758/s13423-016-1142-2

**Published:** 2016-08-25

**Authors:** Max Louwerse, Zhan Qu

**Affiliations:** Tilburg center for Cognition and Communication (TiCC), Tilburg University, Dante Building, Room D 330, Warandelaan 2, 5037 AB Tilburg, The Netherlands

**Keywords:** Form-meaning mappings, Arbitrariness of the sign, Valence, Cross-linguistic approaches, Symbol interdependency

## Abstract

It is assumed linguistic symbols must be grounded in perceptual information to attain meaning, because the sound of a word in a language has an arbitrary relation with its referent. This paper demonstrates that a strong arbitrariness claim should be reconsidered. In a computational study, we showed that one phonological feature (nasals in the beginning of a word) predicted negative valence in three European languages (English, Dutch, and German) and positive valence in Chinese. In three experiments, we tested whether participants used this feature in estimating the valence of a word. In Experiment 1, Chinese and Dutch participants rated the valence of written valence-neutral words, with Chinese participants rating the nasal-first neutral-valence words more positive and the Dutch participants rating nasal-first neutral-valence words more negative. In Experiment 2, Chinese (and Dutch) participants rated the valence of Dutch (and Chinese) written valence-neutral words without being able to understand the meaning of these words. The patterns replicated the valence patterns from Experiment 1. When the written words from Experiment 2 were transformed into spoken words, results in Experiment 3 again showed that participants estimated the valence of words on the basis of the sound of the word. The computational study and psycholinguistic experiments indicated that language users can bootstrap meaning from the sound of a word.

## Introduction

A fundamental question in cognitive science is how language attains meaning. One answer to this question is that meaning must come from outside the language system, because there is no relationship between the sound of a word and its meaning (Glenberg & Kaschak, 2002). This view on arbitrariness of language is of course not new. According to De Saussure (1916), sound and meaning are two abstract sides of a word, and they match each other in an absolutely arbitrary manner (Hockett, 1960). Arbitrariness is indeed important for human languages, because it provides speakers with flexibility and productivity to use and develop the languages (Gasser, 2004).

Recent research in language statistics, however, has shown that words are not as arbitrary as they may seem. Even though word pairs, such as *attic* and *basement*, could occur in any order, a high-low order is significantly more common (Louwerse, 2008), as is the order of *happy* and *sad* in valence words (Hutchinson & Louwerse, 2013) or magnitude (Hutchinson & Louwerse, 2014). We have shown that language users rely on these language statistical patterns in their conceptual processing to bootstrap meaning (Louwerse, Hutchinson, Tillman & Recchia, 2015). The question remains, however, whether the idea that the meaning of a word can be bootstrapped from language statistics beyond word co-occurrence, for instance by using patterns in sound. After all, De Saussure did not focus on linguistic context, but on the word itself when he stated “le signe linguistique est arbitraire” (transl. “The linguistic sign is arbitrary”; De Saussure, 1916, p. 100). Indeed, there seems nothing in the word *happy* that makes it happy, and there is nothing that makes 悲伤 sad. Unless there is.

There is some evidence in the literature that sounds can suggest the meaning of a word. Köhler (1959) showed that English speakers can interpret nonsense word *bouba* as referring to round shapes and *kiki* as referring to angular shapes (Ramachandran & Hubbard, 2001). Taylor and Taylor (1962) used nonsense words with sounds from different languages (English, Japanese, Korean, and Tamil) and asked their native speakers to evaluate the pleasantness of the (nonsense) word, yielding patterns within but not across historically unrelated languages. Auracher, Albers, Zhai, Gareeva, and Stavniychuk (2010) found that for poetic language (poems) the ratio of plosive (e.g. [b], [p]) vs. nasal (e.g. [n], [m]) sounds predicts its emotional tone in German and Japanese. Poems with a high frequency of plosive sounds were more likely to be perceived as positive, whereas a poem with a high frequency of nasal sounds was perceived as more negative. However, Köhler (1959), Ramachandran and Hubbard (2001), and Taylor and Taylor (1962) used nonsense words. Auracher et al. (2010) used poetic (nonliteral) text (beyond single words). That is, evidence against the notion of a word being arbitrarily linked to its meaning should come from real words in non-figurative language.

Monaghan, Chater, and Christiansen (2005) did exactly that by conducting corpus linguistic studies to investigate the potential predictability of lexical categories using phonological cues and found they were able to distinguish nouns from verbs using only such features. Monaghan, Christiansen, and Chater (2007) extended this work by assessing whether phonological cues can assist in determining the syntactic categories of the words in four languages (English, Dutch, French, and Japanese), with English showing a 66.5% accuracy rate, and the other three languages showing around 80% accuracy rates.

The finding that phonological features predict the syntactic category of a word is promising, but the presence of these features leaves the question unanswered whether language users utilize these features in their processing. The current study tested whether phonological features predicted the valence of words and whether language users would rely on these features in their processing. Because there is no evidence that specific phonological features can predict valence, we first conducted a computational linguistic study to investigate the relationship between phonological features and valence across one logographic language (Chinese) and three alphabetic languages (Dutch, English, and German). We thereby applied the principle of parsimony, using the smallest number of features to get the maximum prediction accuracy. We predicted that language users would use those phonological features that predcited valence in the computational study in their valence judgments. We tested this hypothesis in three experiments, focusing on Chinese and Dutch. In Experiment 1, Chinese (and Dutch) participants were asked to quickly decide on whether individual Chinese (and Dutch) words were positive or negative. Experiment 2 was similar to Experiment 1 but switching the languages: Chinese participants responded to Dutch words and Dutch participants responded to Chinese words. In Experiment 3, we replicated the setup of Experiment 2, using sounds rather than written words.

## Study

Words and their valence scores were taken from the Affective Norms for English Words (ANEW; Bradley & Lang, 1999; Warriner, Kuperman & Brysbaert, 2013), a database with valence norms for 13,915 English words. These words were translated into Dutch, German, and Chinese; phonological features of each of the words were determined on the basis of existing phonological databases. Phonological features for these words were derived from the CELEX database (Baayen, Piepenbrock, & Gulikers, 1996). The Dutch and German words were translated from English and their phonological features also were taken from CELEX. For Chinese, the words were translated from English and the Pinyin system of Chinese was used to represent the Chinese words phonetically, and the features were extracted based on this system. The final database consisted of 10,632 Chinese, 10,097 Dutch, 12,676 English, and 8,833 German words and their phonological features. Total counts differed as the matches in the phonological databases differed across the languages. Next, we selected those words with the 20% highest valence ratings (6.1 to 8.53) and the 20% with the lowest valence ratings (1.26 to 4), resulting in 5,453 English, 4,324 Chinese, 4,330 Dutch, and 3,736 German words.

We used 105 phonological features including the 53 features from Monaghan et al. (2007) to assess what features of words were useful to predict the score of valence across the four languages. These features included 1) word level features (e.g., the amount of nasals in the word); 2) proportion of phonemes in a word that belong to the specific sound category (e.g., proportion of nasals); 3) onset of the word (e.g., the amount of nasals in the first syllable of the word), 4) first consonant (e.g., the first consonant of the word is nasal), and 5) vowels (e.g., vowel height). Not all features were present for all languages. For instance, dental (/θ/, /ð/) consonants are only found in English, trills and uvulars only in Dutch and German, whereas tone is found in Chinese. Whereas Dutch, English, and German have near-front, near-back, close-mid, and open-mid vowels, these distinctions do not exist for Chinese.

**Fig. 1:**
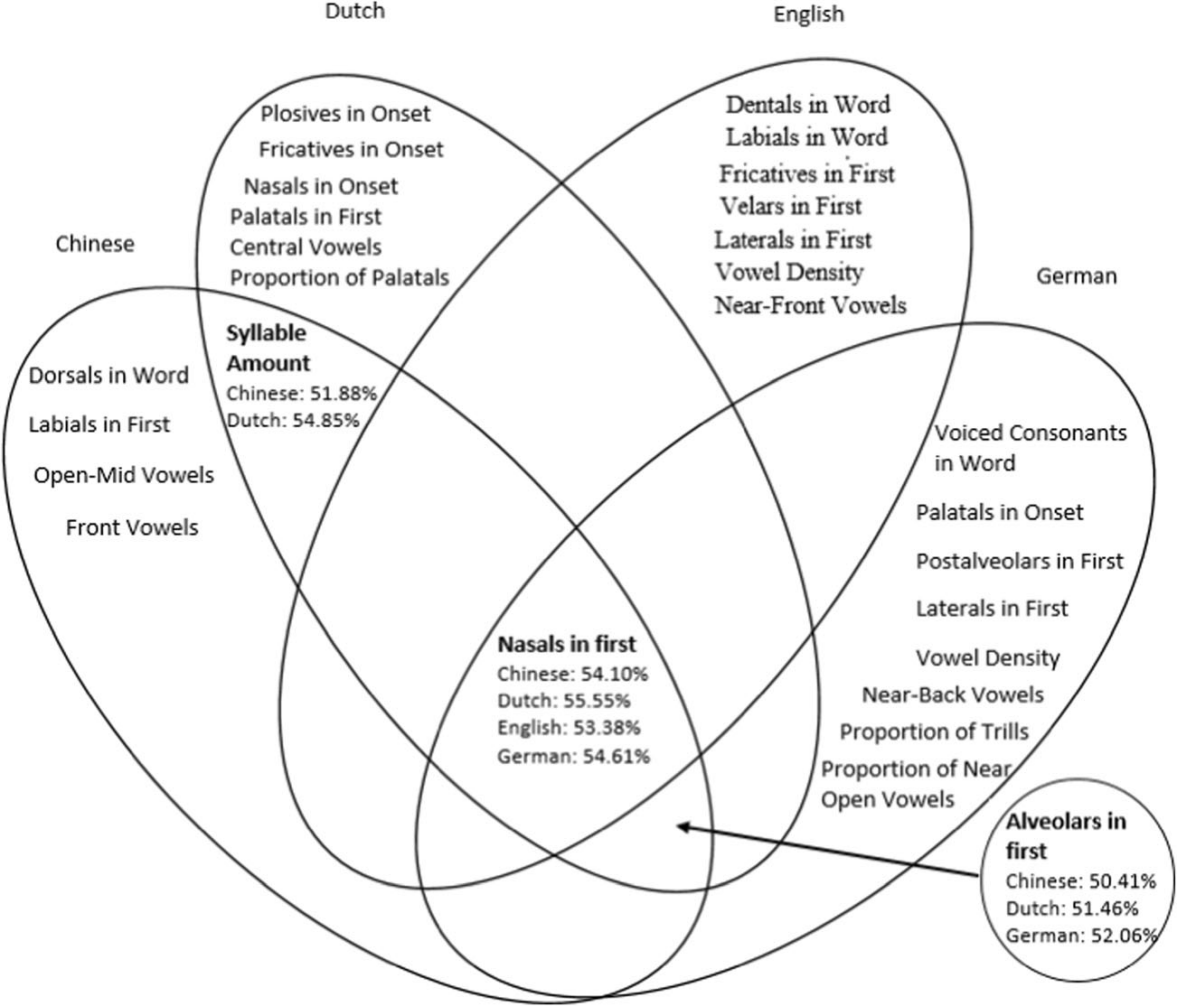
Venn diagram of phonological cues significantly predicting valence, with accuracy of shared phonological features

Next, we used 13 machine learning algorithms (all from Bayes-based, Function-based, Rules-based, Meta-based, and Tree-based algorithm families) to determine which phonological features best explained valence. Because the predictions of these algorithms are very similar, we focused on the Support Vector Machine (SVM) results. We used 70% of the data as a training set and 30% as a test set, and applied tenfold cross-validation to prevent overfitting. Above-chance results were achieved, with 56.78% accuracy for Chinese, 54.66% for Dutch, 55.08% for English, 56.11% for German. Figure 1 shows that the presence of *nasals in the first* position of the word was the best common predictor of valence across the four languages.

Even though nasals in the first position of the word can explain the valence of the word significantly across four languages, these effects are very small. Interestingly, *t*-tests showed that nasals in first position yielded negative valence in Dutch, *t* (9967) = −6.064, *p* < 0.001, English, *t* (12660) = −4.013, *p* < 0.001, and German, *t* (8706) = −4.859, *p* < 0.001, but positive valence in Chinese, *t* (10630) = 2.542, *p* = 0.011. An experimental study can shed light on whether these small effects are nevertheless used by participants in valence judgments. That is, if a word has a neutral valence rating, do participants nevertheless show patterns matching the computational linguistic study in valence judgements, and if this is the case, do speakers of Germanic and Chinese languages do this in opposite ways?

## Experiment 1

Experiment 1 investigated whether language users used the phonological feature (nasals in first position) found in the computational study in their processing of the valence of words. Because of the access to participant populations and because of their opposite results—valence positively correlated with nasals in Chinese, but negatively with Dutch—we chose Chinese and Dutch as the languages in Experiments 1–3. We predicted that Chinese speakers were more likely to consider written words starting with nasals as more positive, whereas Dutch speakers were more likely to consider written words starting with nasals as more negative in valence.

### Method

#### Materials

We randomly sampled 100 Dutch and 100 Chinese words from the ANEW database that had a neutral valence rating (4.5-5.5 on a scale of 1-9), such that 50 words had nasals-first structure and 50 words did not have this structure. Details on the Chinese and the Dutch words are presented in Table 1.

**Table 1:** Overview of the Chinese and Dutch words in means (standard deviations)

	Chinese		Dutch	
	Nasals in first position	Nonnasals in first position	Nasals in first position	Nonnasals in first position
Valence	5.01 (0.07)	5.08 (1.28)	5.11 (0.26)	4.94 (0.12)
Log frequency	16.2 (1.91)	15.03 (1.93)	13.55 (1.72)	12.77 (0.96)
Number of syllables	2.06 (0.24)	2.24 (0.43)	2.06 (0.87)	2.12 (0.72)
Number of phonemes	4.14 (0.95)	5.04 (1.28)	6.44 (2.41)	6.54 (2.19)

#### Participants

Fifty native speakers of Chinese (20 females) and 50 native speakers of Dutch (23 females) were asked to volunteer in this experiment. Participants were recruited on the Tilburg University campus, where the default language that is spoken is English.

#### Procedure

Participants were seated in front of a laptop computer (MacBook pro, 13” screen) and were asked to evaluate each word as fast as possible on its valence by clicking positive (*z* key) or negative (*m* key) and were told there were no correct answers. Words were presented individually in the center of the screen (Arial, font size 12) in a black font against a white background. Chinese speakers saw the word in the Pinyin system of Chinese as well as in Chinese characters (simplified Chinese was used as participants came from mainland China). After participants responded to a word, a new word appeared on the screen. If participants did not respond within 2 seconds, a new word automatically appeared with no participant answer being recorded.

### Results and discussion

For the Chinese participants, we found 0.7% of the items not being responded to, because participants did not make a decision within the allocated time of 2 seconds. Of the items, participants responded to 60.5% of the words as having a positive connotation. For the Dutch participants, a similar pattern emerged: 0.8% of the items missing and participants responded to 59% having a positive connotation. The speed with which the Chinese participants responded was similar to the response time of the Dutch participants, *M* = 760 milliseconds (*SD* = 270) and *M* = 770 milliseconds (*SD* = 280) respectively.

**Fig. 2:**
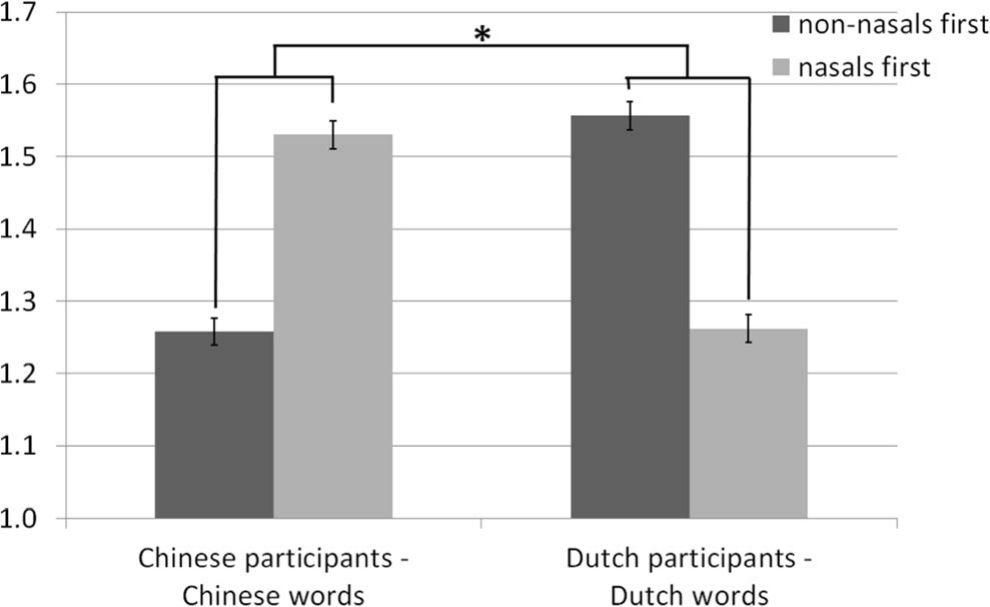
Valence ratings (1 = negative valence, 2 = positive valence) for written words in native languages of Dutch and Chinese participants

No significant difference was found between Chinese and Dutch participants, *F*(1, 196) = 0.65, *p* = 0.42, η_p_^2^ = 0.003, and no effect was found between the ratings based on the nasal-first feature, *F*(1, 196) = 0.35, *p* = 0.56, η_p_^2^ = 0.002. However, an interaction was found between the native language speakers and the nasal-first pattern, *F*(1, 196) = 225.91, *p* < 0.001, η_p_^2^ = 0.54, in the expected direction, such that Chinese participants rated nasals-first words as being more positive, whereas Dutch participants rated nasals-first words as being more negative (Fig. 2). Indeed, the ratings of Chinese words in the experiment were positively correlated to nasals in first, *r* = 0.738, *p* < 0.001, and valence of valence-neutral Dutch was negatively correlated to nasals in first, *r* = −0.505, *p* < 0.001. However, to avoid judgments being explained by other variables than the nasals in first position, we conducted two experiments with words unknown to the participants.

## Experiment 2

Experiment 2 extended Experiment 1 by not taking the Chinese but the Dutch words for the Chinese participants (and the Chinese words instead of the Dutch words for the Dutch participants). We predicted the same patterns as in Experiment 1 with speakers of Chinese speakers judging nasals in the beginning of the written word as more positive, with an opposite result for speakers of Dutch.

### Method

#### Materials

The same words with neutral valence ratings as in Experiment 1 were used. To make the Chinese words readable for Dutch speakers, Chinese characters were now presented in the Pinyin system of Chinese.

#### Participants

Fifty native speakers of Chinese (19 females) and 50 native speakers of Dutch (21 females) were recruited in the same way as in Experiment 1. No participants from Experiment 1 participated in Experiment 2. None of the Dutch participants spoke Chinese, and none of the Chinese participants spoke Dutch.

#### Procedure

The procedure was identical as in Experiment 1. Participants were asked to evaluate each word as fast as possible on its valence by clicking positive or negative. Participants were told explicitly that there were no correct answers. Note that participants would not be able to predict a correct answer, as none of the (foreign) words were familiar to the participants.

### Results and discussion

For the Chinese participants, 0.3% of the data was missing and the results showed that the valence ratings for words they could not know approached chance level (52.6% rated positive and 47.4% rated negative). For the Dutch participants, these findings were not different: 0.6% of the data were missing and participants rated 47.3% as positive and 52.7% as negative. As before, the time within which participants responded was similar between the two language groups, for Chinese (*M* = 780 milliseconds, *SD* = 300) and Dutch (*M* = 730 milliseconds, *SD* = 420).

**Fig. 3:**
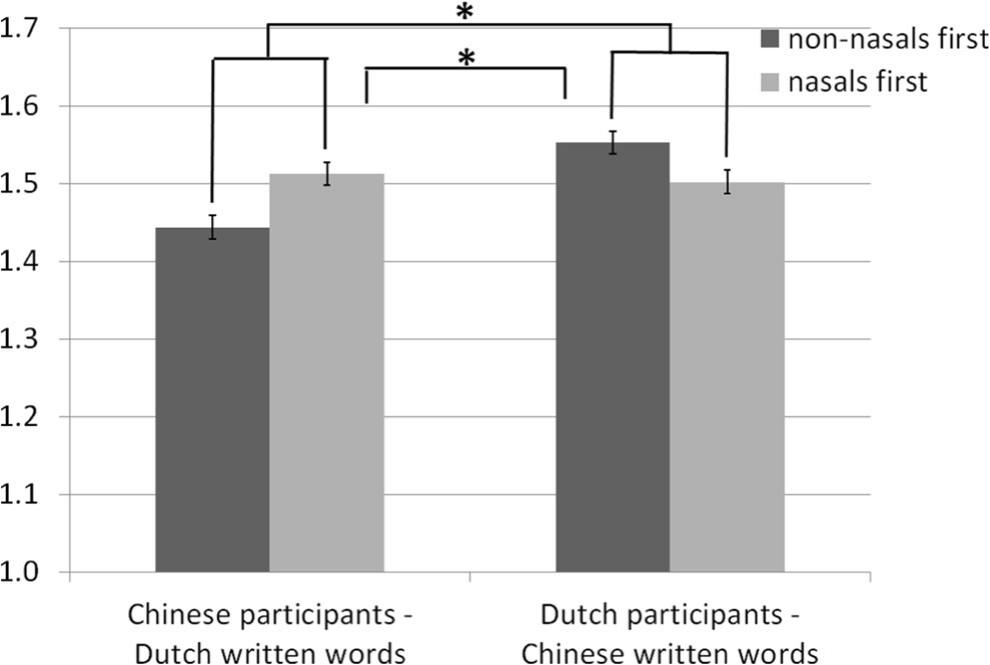
Valence ratings (1 = negative valence, 2 = positive valence) for written words in opposite languages (Chinese participants reading Dutch words and Dutch participants reading Chinese words)

As in Experiment 1, no significant difference was found for the way nasals first were rated across both language groups, *F*(1, 147) = 1.09, *p* = 0.29, η_p_^2^ = 0.005. However, contrary to Experiment 1, a significant difference was found in the ratings between the Dutch and the Chinese participants, *F*(1, 147) = 30.83, *p* < 0.001, η_p_^2^ = 0.126, with higher positive ratings for the Dutch participants. We do not have an explanation for this difference, other than that the Dutch participants rated items more positively. Importantly, as in Experiment 1 an interaction was found between language and the nasals-first pattern, *F*(1, 147) = 45.54, *p* < 0.001, η_p_^2^ = 0.176, yielding the same result as in Experiment 1, except that this time participants had nothing to rely their valence ratings on, other than the graphemes of the word representing the sounds (Fig. 3).

The problem with Experiment 2 is that participants had to make judgments based on graphemes. Even though we could argue that readers processed the graphemes as phonemes in the reading of the word, this is not at all certain. To adequately determine whether the phonemes of a word support predicting its meaning, a spoken word presentation is needed.

## Experiment 3

Experiment 3 replicated Experiment 2 using sound rather than printed words. In addition, in Experiment 1 and 2 we recruited Chinese participants at a Dutch university campus where the main language spoken is English. In Experiment 3, Chinese participants were recruited in China. We predicted the same patterns as in Experiment 2 with Chinese speakers rating valence more positive for nasals in the beginning of the spoken words and Dutch speakers rating valence more negative for nasals in the beginning of the spoken words.

### Method

#### Materials

The same words from the previous two experiments were used. The words were pronounced by text-to-speech tools. For Dutch, Ivona (www.ivona.com) was used, an engine that did not provide Chinese. For Chinese, Neospeech (www.neospeech.com) was used, an engine that did not provide Dutch. For both languages, the male voice was selected. The quality and naturalness of the voice recordings were checked by native speakers of Dutch and Chinese.

#### Participants

Ninety-seven native speakers of Chinese (46 females) and 88 native speakers of Dutch (41 females) volunteered in this experiment. None of the participants had participated in the previous two experiments. Chinese participants were recruited on the campus of Baotou Medical College in Baotou, China and did not know any Dutch. Dutch participants were recruited on the Tilburg University campus and did not know any Chinese.

#### Procedure

In general, the setting of this experiment was identical to the previous two experiments, except that participants were provided with audio recordings of the words. Participants were seated in front of a desktop computer and listened to each word through headphones. They had 2.5 seconds to make their judgements. If they did not respond within 2.5 seconds of the offset of the word, the next audio stimulus was presented.

### Results and discussion

Because audio files had to be loaded and may have malfunctioned, the number of missing values was considerably higher than in the previous two experiments, with Chinese participants missed 29.1% of the items. Their valence ratings were 55.7% positive and 44.3% negative for the valence-neutral words. For the Dutch participants, 30.3% of the items were missing, and participants were 54.3% positive and 45.7% negative in their ratings. The response times were also higher than in the previous experiments, for Chinese (*M* = 2.09, *SD* = 0.54) and Dutch (*M* = 1.92, *SD* = 0.61).

**Fig. 4:**
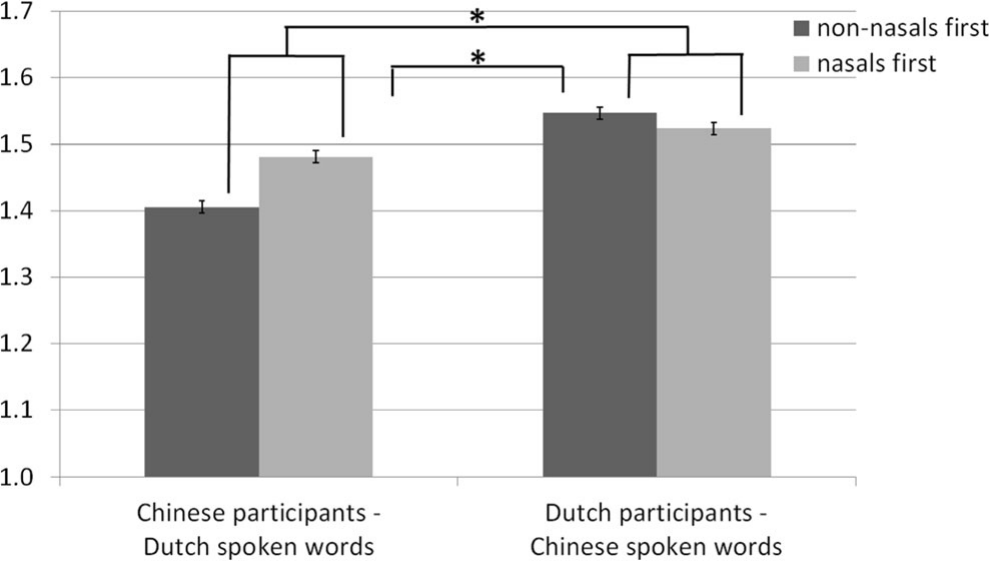
Valence ratings (1 = negative valence, 2 = positive valence) for spoken words in opposite languages (Chinese participants hearing Dutch words and Dutch participants hearing Chinese words)

No significant difference was found for the nasal variable, *F*(1, 363) = 3.00, *p* = 0.084, η_p_^2^ = 0.008. As in Experiment 2, a significant difference was found between Chinese and Dutch, *F*(1, 363) = 33.734, *p* < 0.001, η_p_^2^ = 0.085. More importantly for the purposes of this study, an interaction was found between the languages and the nasal-first pattern, *F*(1, 363) = 10.127, *p* = 0.002, η_p_^2^ = 0.027, again in the expected direction (Fig. 4).

## General Discussion

The findings presented in this paper show that phonological features can predict the valence of the word across three Germanic languages and Chinese. In addition to computationally showing that phonological features predict the valence of a word, we tested whether language users rely on these features. In a SVM classification analysis, we found that nasals in the beginning of the word yielding more positive ratings for Chinese but more negative valence ratings for Germanic languages. In Experiment 1, we asked Chinese and Dutch participants to rate the valence of valence-neutral words and found that Chinese and Dutch speakers relied on the patterns that were extracted from the computational study. Experiments 2 and 3 reversed the languages so that participants looked at words they did not know, as written words (Experiment 2) and spoken words (Experiment 3). The results from all three experiments showed that language users relied on nasals in the first position to estimate the valence of the word, with native speakers of Chinese judging nasals-in-first-position as being more positive and Dutch speakers rating those more negative. We can only speculate why nasals in the first position yield these opposite results in Dutch and Chinese. One possibility is that the nasal pattern can be found in negative words in Germanic languages, such as *no*, *negative*, *night*, *minus*. However, the generalizability of this pattern requires further investigation.

The findings reported here clearly do not deny the claim that there is not a one-to-one relation between the sounds in a word and its meaning. Even though the results were significant and consistent across the four studies, the effects were small. However, the results warrant reconsidering a strong arbitrariness claim that suggests no meaning can be extracted from the sound of a word.

The findings presented here are in line with what we have claimed for first-order (Hutchinson & Louwerse, 2013) and higher-order (Recchia & Louwerse, 2015) co-occurrences. Language users translate prelinguistic conceptual knowledge into linguistic conceptualizations, so that as a function of language use, language statistical patterns are encoded in language (Louwerse, 2008). Consequently, language users can rely on statistical linguistic cues in their meaning estimates at least to generate good-enough representations. What we found for co-occurrences between words we can now extend to phonological features within words.

The effect sizes may seem small and therefore these findings may seem negligible. However, let us assume the general linguistic context of a word can predict the valence of a word (Recchia & Louwerse, 2015) and so can the immediately preceding words (Hutchinson & Louwerse, 2013). The current study has shown that nasals in the first position of the word predict the valence only with approximately 55% accuracy (Event A). Let us assume that using first-order co-occurrence frequencies the probability of predicting the valence of a word correctly is also only 55% (Event B). Now, let us assume that a language user does not know any Chinese, but hears the word pair 快乐 - 悲伤. On the basis of the sound only, the language user can predict with 55% accuracy that快乐 is happy and 悲伤 is sad either on the basis of phonological features or first-order co-occurrences. However, combined valence can be predicted with 80% accuracy (i.e., P (A U B)). Obviously, this is merely an illustration, as it does not include higher-order co-occurrences and excludes any symbol grounding. The estimates for an experienced language user are therefore likely to be considerably higher than 80% accuracy when other factors are included. However, with limited symbol grounding, language statistics allows for bootstrapping meaning throughout the network of language. This is no different for associations between words, as it is for the phonological features of a word.
